# The functional and clinical outcomes of exercise training following a very low energy diet for severely obese women: study protocol for a randomised controlled trial

**DOI:** 10.1186/s13063-016-1232-5

**Published:** 2016-03-08

**Authors:** Clint T. Miller, Steve F. Fraser, Steve E. Selig, Toni Rice, Mariee Grima, Nora E. Straznicky, Itamar Levinger, Elisabeth A. Lambert, Daniel J. van den Hoek, John B. Dixon

**Affiliations:** School of Exercise and Nutrition Sciences, Deakin University, 221 Burwood Highway, Burwood, VIC 3125 Australia; Clinical Obesity Research Laboratory, Baker IDI Heart and Diabetes Institute, Melbourne, VIC Australia; Human Neurotransmitters and Clinical Obesity Research Laboratory, Baker IDI Heart and Diabetes Institute, Melbourne, VIC Australia; Clinical Exercise Science Research Program, Institute of Sport, Exercise and Active Living (ISEAL), Victoria University, Melbourne, VIC Australia

**Keywords:** Obesity, Exercise, Body composition, Fitness, Very low energy diet

## Abstract

**Background:**

Clinical practice guidelines globally recommend lifestyle modification including diet and exercise training as first-line treatment for obesity. The clinical benefits of exercise training in adults with obesity is well-documented; however, there is no strong evidence for the effectiveness of exercise training for weight loss in class II and class III obesity. The purpose of the randomised controlled trial described in this protocol article is to examine the effect of exercise training, in addition to a very low energy diet (VLED), in clinically severe obese women for changes in body composition, physical function, quality of life, and markers of cardiometabolic risk.

**Methods/Design:**

Sixty women, aged 18–50 years with a body mass index (BMI) greater than 34.9 kg.m^2^ and at least one obesity-related co-morbidity, will be recruited for this 12-month study. Participants will be randomised to either exercise plus energy restriction (*n* = 30), or energy restriction alone (*n* = 30). All participants will follow an energy-restricted individualised diet incorporating a VLED component. The exercise intervention group will also receive exercise by supervised aerobic and resistance training and a home-based exercise programme totalling 300 minutes per week. Primary outcome measures include body composition and aerobic fitness. Secondary outcome measures include: physical function, cardiometabolic risk factors, quality of life, physical activity, and mental health. All outcome measures will be conducted at baseline, 3, 6 and 12 months.

**Discussion:**

Previous research demonstrates various health benefits of including exercise training as part of a healthy lifestyle at all BMI ranges. Although clinical practice guidelines recommend exercise training as part of first-line treatment for overweight and obesity, there are few studies that demonstrate the effectiveness of exercise in class II and class III obesity. The study aims to determine whether the addition of exercise training to a VLED provides more favourable improvements in body composition, physical function, quality of life, and markers of cardiometabolic risk for women with clinically severe obesity, compared to VLED alone.

**Trial registration:**

Australian New Zealand Clinical Trials Registry (ACTRN12611000694910). Date registered: 4 July 2011

## Background

Around one quarter of Australian and one third of US adults are classified as obese [1, 2]. Further, there is evidence of an acceleration in the prevalence of those approaching class III obesity (body mass index, BMI ≥40 kg.m^2^), compared to overweight and class I obese individuals (BMI 25–34.9 kg.m^2^) [2–4]. Individuals in classes II (BMI 35–39.9 kg.m^2^) and III (BMI ≥40 kg.m^2^) obesity are at a higher risk of developing metabolic and cardiovascular disease, and disability compared to those with overweight and class I obesity [3, 5, 6]. This translates to clinical health care costs that are twice that of healthy-weight individuals [7].

Diabetes mellitus, cardiovascular disease, sleep apnoea, dyspnoea, mental illness, musculoskeletal pain and disorders all impact negatively on an individual’s capacity to perform activities of daily living and are more prevalent in obese individuals [8–11]. Reduced capacity for activities of daily living may occur prior to the development of these conditions and may be related to adverse metabolic and biomechanical changes associated with obesity [12, 13]. Obese individuals often experience a vicious cycle of low exercise capacity, physical disability and breathlessness leading to physical inactivity, and further weight gain [14], loss of physical function [15, 16], and frailty [13, 17, 18].

Regular aerobic or resistance exercise training influences physical fitness and functional capacity through improvements in muscular strength, power, endurance, and cardiorespiratory and vascular fitness. Aerobic exercise training specifically facilitates the improvement in central and peripheral cardiorespiratory, vascular and metabolic function, while resistance training improves muscular endurance, strength, power and hypertrophy [19]. Obese adults have been reported as having a blunted response to exercise training when exposed to the same training stimulus as their healthy-weight peers [20]. The addition of specific exercise training to energy restriction in obesity may, in addition to changes in physical fitness, confer favourable body composition outcomes but the evidence in clinically severe obesity is limited [21–23].

Lean mass is integral to the long-term maintenance of metabolic rate, core body temperature, skeletal integrity, muscle strength, functional capacities [24, 25], and the prevention of sarcopenic obesity later in life [26]. During weight loss a greater proportion of lean mass is lost compared to when weight is regained [27, 28]. The resulting lean mass deficit and continued lack of physical activity associated with ageing may lead to increased risk of physical disability later in life [29, 30]. Exercise in addition to weight loss improves physical function to a greater extent than energy-restricted weight loss alone [23]. It is not clear whether aggressive dietary weight loss with the addition of exercise training is any more beneficial, compared with dietary weight loss alone, in clinically severely obese individuals. Although exercise training during an energy-restricted diet slows the loss of lean mass [22, 23, 31, 32], exercise training may be more important as a method to enhance long-term maintenance of weight loss [33–35].

Clinical practice guidelines recommend exercise training together with energy restriction as a first-line intervention for obesity, without strong evidence of the effectiveness of exercise training for weight loss in class II and class III obesity [36–40]. Even if exercise training proves to not be effective for weight loss in severely obese individuals, there are other important benefits to explore for them. Exercise training alone has been reported to improve glucose regulation and slow the progression of type 2 diabetes [41–52], reduce systolic and diastolic blood pressure (BP) [53–60], and reduce total cholesterol, low-density lipoprotein (LDL) cholesterol, and triglycerides [59, 61, 62], independent of weight loss in overweight and obese adults. Improvements have also been noted for symptoms of depression [63–69] and quality of life [70, 71] in obese individuals. Individuals with severe obesity may respond differently to these outcome measures than overweight individuals. Therefore, the purpose of the randomised controlled trial described here is to examine the unique effect of exercise training, in addition to a very low energy diet (VLED), in women with severe obesity for changes on body composition, physical function, quality of life, and markers of cardiometabolic risk.

## Methods

### Design

**Fig. 1:**
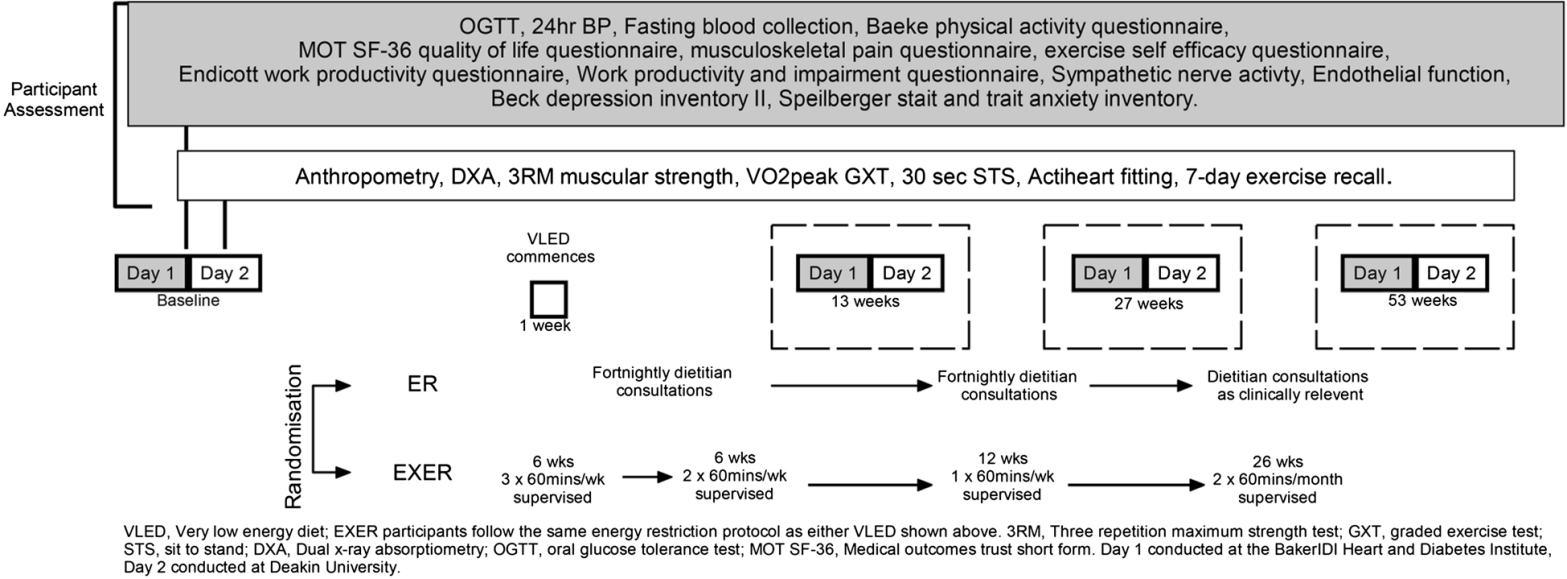
Trial flow chart outlining participant testing and intervention schedule

This randomised clinical trial is summarised in Fig. 1. The study has been approved by the Deakin University Human Research Ethics Committee (reference, 2011–154), the Alfred Human Research Ethics Committee (reference, 330/11) and is registered with the Australian New Zealand Clinical Trials Registry (ACTRN12611000694910). The trial will be conducted in accordance with the Helsinki Declaration [72] and reporting of the study will adhere to the Consolidated Standards of Reporting Trials (CONSORT) guidelines [73]. Following the initial screening process, the women will be randomised into either the energy restriction alone group (ER) or the energy restriction plus exercise intervention group (EXER).

### Participants

Women aged 18–50 years, with a BMI greater than 34.9 kg.m^2^ and at least one obesity-related co-morbidity, will be recruited. Volunteers will be excluded if they report serious unstable cardiovascular conditions, type 1 diabetes, active musculoskeletal conditions that limit exercise participation, pregnancy, planning pregnancy in the next 12 months or breastfeeding, weight loss greater than 5 kg in the past 3 months, currently using weight loss medication or having undergone weight loss surgery in the past, currently using medication which significantly influences weight (antipsychotic drugs, some corticosteroids, anticonvulsant drugs, Loperamide, Buscopan, insulin), and currently undertaking more than 150 minutes of moderate to vigorous intensity exercise each week. Each participant will receive written and verbal explanations about the nature of the study and the testing procedures. Those participants who choose to participate must provide informed consent by signing a participant information and consent form and consult with their own general practitioner to obtain a medical clearance prior to formally being accepted to participate.

### Recruitment

Participants volunteering for the study will be recruited through Deakin University, Monash Medical, and Baker IDI Heart and Diabetes Institute internal email networks. Advertisements will be placed in one large locally subscribed weekend newspaper and other local area newspapers. Study details and contact details will be listed on the Baker IDI Heart and Diabetes volunteer webpage, promoted on social media (Facebook and Twitter). One-page information flyers will be given to medical practices in the local area after meeting with practice nurses and dietitians, and flyers placed in medical practice waiting rooms. Interested volunteers self-initiate contact with the research team via email or phone. Interested volunteers will be screened against the inclusion criteria prior to being given the participant information and consent form, and medical clearance form.

### Sample size

The number (*n* = 60) of participants to be recruited for this study has been determined based on previous evidence, plus a projected drop-out rate of 25 %, and provide adequate statistical power for the two co-primary outcome measures of VO_2peak_ (*n* = 20, *α* = 0.05, power 0.9 [74]) and lean mass (*n* = 23, SD of 1.7, *α* = 0.05, power 0.8 [75]). Secondary outcome measures for traditional markers of cardiometabolic health including systolic and diastolic blood pressure, total cholesterol, LDL cholesterol, insulin sensitivity; and measures of physical activity require fewer participants to reach statistical power (range *n* = 8–14, *α* = 0.05, power 0.8).

### Randomisation

Participants will be randomised individually by a researcher external to the project and will have no contact with the participants prior to or during the trial. This researcher will have no intellectual or personal investment into the study design or outcomes. The women will be randomised into either (ER) or (EXER) groups using sealed opaque envelopes in blocks of six. The envelopes will be stored in a locked cabinet within the independent researcher’s office. Each envelope will contain a single sheet of paper identifying the group allocation. The sheet of paper will be folded at least twice to reduce chance of transparency. One envelope will be chosen at random from the stack of six envelopes containing three of each group allocation (EX and EXER) reduced to a single envelope; this is then combined with the next group of six envelopes to reduce the chance of predictability. The independent researcher is provided with a participant identification number and completes randomisation once the prospective participant provides the study investigators (CM, TR, DV or JD) with a medical clearance from their own general practitioner, preliminary participant screening, and completes all of the baseline measures to ensure safety and suitability for study participation. Participants, and researchers involved in delivering participant interventions will not be blinded to group allocation. Some of these researchers (CM, DV, TR) will also be involved in study co-ordination and data collection.

### Study interventions

#### Energy restriction

All participants in ER and EXER follow the same structured Optifast® clinical treatment protocol. In summary, the participant will meet with an accredited practicing dietitian who forms part of the research team every fortnight for the first 12 weeks. This will be continued as clinically relevant for the remaining 9 months. The VLED consists of four phases (intensive, 450–680 kcal; transition, 800–880 kcal; maintenance, 1000–1400 kcal; and stabilisation, 1200 kcal; phases). The phases and the extent of energy restriction of the protocol are not fixed, and will be modified as clinically relevant depending on the participant’s ability to manage with the diet, weight gain or loss, or the participant’s change in health circumstances. It is expected that not all participants will feel comfortable following the Optifast® protocol for the entire 12 months despite encouragement and support and, therefore, the dietitian will work with the participant to create a suitable energy-restricted diet as per standard clinical practice. All participants begin with an intensive phase for up to 12 weeks, moving into the transition phase for weeks 13–18 which incorporates one whole food meal, completing weeks 19–22 in the maintenance phase to introduce two whole food meals per day, and the remaining 6 months in the stabilisation phase where most meals will consist of whole food. As part of standard clinical care, the study dieticians will collect 4-day diet records at baseline and at each follow-up period and, when required, use the 24-hour dietary recall for tracking participant adherence and adjust the dietary plan.

#### Exercise training

EXER follow a standardised progressive exercise programme led by accredited exercise physiologists or a student exercise physiologist supervised by an accredited exercise physiologist. The exercise intervention consists of a supervised exercise programme that is complemented by a home-based exercise routine. The supervised exercise training will commence the same week as the VLED and consists of the following:

- 3 × 1 hour supervised exercise sessions per week (weeks 1–6)
- 2 × 1 hour supervised exercise session per week (weeks 7–12)
- 1 × 1 hour supervised exercise session per week (weeks 13–26)
- 2 × 1 hour supervised exercise sessions per month (weeks 27–52)

The exercise training will consist of a progressive moderate intensity aerobic conditioning and resistance training programme using a variety of machines and free weights. Each training session lasts 60 minutes and will consist of 5 minutes’ warm up, 45–50 minutes of conditioning, and 5 minutes of cool-down and stretching. The conditioning component consists of approximately 20–30 minutes of aerobic training (12–16 on Borg’s Ratings of Perceived Exertion (RPE) scale, 60–80 % heart rate reserve (HRR)) followed by 20–30 minutes of resistance exercise (one to three sets of eight to ten repetitions). The resistance exercise involves six to ten different, predominantly multi-joint exercises for the upper and lower extremities at an intensity of two repetitions below volitional fatigue, with a 2-second concentric and eccentric contraction speed. Exercise prescription followed clinical judgement and the American College of Sports Medicine’s (ACSM’s) guidelines [76] to ensure fitness improvement and programme compliance. Once participants complete three sets of ten repetitions with correct technique, for two consecutive training sessions, the resistance will be increased. The cardiorespiratory fitness training progress from 40–60 % HRR in the first 5 weeks to 60–80 % HRR for the remainder of the programme. The mode of aerobic exercise varies depending on participant’s choice; however, weight-bearing exercise such as walking will be encouraged as one of the principle exercise modes.

The exercise physiologists will follow a structured resistance training programme with eight core exercises that will include: leg press, squats, bench press, seated row, lateral pull down, shoulder press, biceps curl and triceps kickbacks.

The exercise physiologists will keep a record of attendance, exercises undertaken and progress achieved by participants. Any adverse events or adverse signs and symptoms will be documented including feelings of general fatigue, soft tissue soreness, light-headedness, colds and flu and injury or illness. The reason for non-attendance will also be included on the exercise programme card. ‘Make up’ sessions will be made available where possible during the same training week or the following week.

The participants will be assisted in the development of a home-based exercise programme that will gradually replace the supervised exercise sessions. This is a transfer to a self-management model. The programme will consist predominantly of cardiorespiratory exercise devised around each individual’s access to facilities and equipment. Participants will also be encouraged to increase incidental physical activity. Participants will progressively increase the weekly hours and frequency of home-based exercise during the period of the study so that all participants aim to be exercising most if not all days of the week. The participants will set a goal of exercising at a moderate intensity for 200 minutes per week in week 1, progressing to 300 minutes by week 6 and sustaining this level of exercise participation for the remainder of the 12-month intervention to assist with developing a regular exercise habit conducive for weight maintenance [77–79]. The purpose of the exercise physiologist is to provide advice, guidance and support for the prescription of exercise, and at no time will provide dietary advice to the participants. Exercise physiologists will advise participants to discuss dietary questions with the study dietitians.

Participants randomised to the energy restriction only group will be encouraged to exercise at study commencement and during the dietary consultations along with general lifestyle advice, but will not be provided with any other specific guidance or follow-up support. These participants will not be discouraged from changing their exercise habits.

### Measures

Outcome measures will be obtained from participants at baseline, 3, 6, and 12 months. The series of tests at each location is shown in Fig. 1. Trained research staff will adhere to the standardised procedures for all data collection ensuring consistency in (1) order of tests, (2) use the same equipment for each test, and (3) management and storage of data collected.

### Primary outcome measures

#### Peak aerobic power

Peak aerobic power will be assessed using a symptom-limited graded motorised treadmill (h/p/cosmos Quasar DE83365, Nussdorf-Traunstein, Germany) test suitable for those with low exercise tolerance. Participants warm up at a comfortable walking pace at 0 % gradient and speed is then adjusted gradually until RPE (6–20 Borg point scale) of 8/20 is achieved. Participants will be coached and counselled on the RPE scale during the rest period and as they progressively reach the test speed. The speed will then remain constant for the duration of the exercise test. During the test period the gradient is increased by 2 % per minute until the participant subjectively reports a 17 (very hard) on the Borg rating of perceived exertion scale (RPEpeak = 17). The test will be terminated earlier if adverse signs and symptoms arise. The test will be terminated at an RPE of 17/20 to avoid adverse signs and symptoms rather than reaching maximum volitional fatigue, which may increase the risk of adverse events, or increased risk of withdrawal from the study in this high-risk population. Using RPE for test termination is a valid method of measuring peak aerobic power [80–82]. Expired respiratory gases will be collected through a breath-by-breath pneumotach system (Innocor Innovision version 6.15, Glamsbjerg, Denmark). The Innocor unit will be calibrated before each test according to the manufacturer’s guidelines. The breath-by-breath data will be integrated for each 15-second interval and the mean values for VO_2_, VCO_2_ and ventilation (VE) used for that interval. Heart rate (HR) will be measured at rest and during the graded exercise test using a 12-lead electrocardiogram (ECG) (Mortara, X-Scribe II, Milwaukee, WI, USA). Heart rhythm and other ECG characteristics will be continuously monitored from this 12-lead system. Pulse oximetry (NONIN Medical 2120, Plymouth, MN, USA) will be recorded at the end of each minute during the test and recovery. Participants will be asked not to consume caffeine, alcohol or tobacco for a minimum of 2 hours prior to the exercise test. Resting pre-exercise and post-exercise blood pressure will be measured using a manual sphygmomanometer and stethoscope.

### Muscular strength

Maximal muscular strength will be determined using a three repetition maximum (3RM) [76] for bench press, supine leg press, and seated row using standard resistance training equipment. The participants will be instructed and allowed to practice correct lifting and breathing techniques for each exercise prior to the test and will complete ten repetitions of the exercise at a low to moderate load. This also serves as a specific warm up to the exercise test. Participants will be asked to self-select an estimated starting weight which will then be increased gradually until only three repetitions are possible. The progressive increase in load ranges from 1–10 kg for bench press, 2–10 kg for leg press and 1–9 kg for seated row. The rest period between attempts is set to 90–120 seconds or until fully recovered. The bench press will be performed using a Smith machine (BodySolid, Powerline PSM144X, Forest Park, IL, USA). The seated row will be completed using a dual pulley (Nautilus tower pulley system, F3ATFS, Independence, VA, USA) with a V-pulldown/row bar. The pulley height will be set in line with the participant’s xiphoid process and feet placed on a step to ensure a full arm length distance from the pulley. The back and shoulders will rest on the back of the upright bench to avoid the use of momentum. The leg press (Synergy Omni leg press S-31-OPD, QLD, Australia) will be performed in supine with the seat lowered until the knee reaches 90°. For all strength tests, the machine settings in relation to anatomy will be recorded and replicated in subsequent tests. Total muscle strength will be calculated as the sum of the three strength measures. Relative strength will be calculated by dividing total strength by body mass in kilograms.

### Body composition

Body composition and total body bone mineral density will be assessed using a dual energy X-ray absorptiometry (DXA) scanner (GE Lunar Prodigy Pro, Madison, WI, USA) with software version 12.1 to assess total and regional body fat mass, lean mass, and total body bone mineral content and density. Prior to each assessment standard manufacturer procedures for quality assurance and quality control will be performed. All participants will be wrapped using a light sheet to ensure the entire body remains within the scan area. Due to the limited scanning width of the DXA, modifications to the scan will be completed and replicated during subsequent scans. Participants will be aligned with the right hand side of their body against the right edge of the scan field. In some instances due to the width of obese participants, the left upper extremity may fall outside of the scan range. For these participants, the left upper extremity will be replaced by the right upper extremity. For instances where portions of the trunk lie outside of the scan area, half body scans will be completed of the right side and multiplied by two. The maximum static load limit of the DXA at the Deakin University laboratory is 159 kg. Participants above this weight will be scanned at the Alfred Hospital using a DXA scanner (GE Lunar iDXA, Madison, WI, USA) with an upper weight limit of 200 kg. For all participants, follow-up testing will be replicated to be consistent with that of the baseline test, including body position, calculation, and equipment.

### Secondary outcome measures

#### Body mass index

Weight will be measured using calibrated scales (SECA 708, Hamburg, Germany), weighing to the nearest 0.01 kg. Height will be determined using a standard calibrated stadiometer (Holtain Limited, Crymych, Pembrokeshire, UK) measured to the nearest 0.01 metres with the participant standing in the anatomical position. BMI will be calculated using the following formula: weight (kg)/height (m^2^). Three measurements will be taken and the mean of the two closest measurements used.

### Waist circumference

Waist circumference will be measured using a steel tape measure with the participant in the standing position. The tape will be placed horizontally around the participant’s waist immediately above the iliac crest according to the NHANES III procedure [83]. Three measurements will be taken and the mean of the two closest measurements will be used.

### Hip circumference

Hip circumference will be measured using a steel tape measure with the participant in the standing position. The tape will be placed horizontally around the widest part of the hips [84]. Three measurements will be taken, and the mean of the two closest measurements used.

### Sit to Stand test in 30 seconds

The Sit to Stand test in 30 seconds (STS-30) as described previously [85] will be performed as a functional weight-bearing performance test for the lower body and a test for lower limb muscular endurance. The test commences following a demonstration of technique and cueing from the researcher and is practised by the participant for up to ten repetitions with feedback on technique. The participant then rests for up to 3 minutes or until fully recovered before commencing the 30-second test. The test commences in the seated position and the participants are instructed to hold their arms across their chest for the test duration. The participant will be required to sit back on the chair but not collapse back into the chair prior to the next repetition. If the participant is more than half way up at the end of 30 seconds then the repetition will be rounded up. The chair will be set at the height of the popliteal crease and recorded to replicate in later tests.

### Objective assessment of physical activity and sedentary behaviour

Participants will be fitted with an Acti-heart® heart rate and activity monitor that will be worn for 7 days as described previously [86, 87]. The monitor is attached by two standard electrodes on the chest, placed immediately lateral to the sternum at the second intercostal space with the second electrode attached on the same horizontal level as far lateral as possible. The area is cleaned with a 73 % ethanol swab prior to electrode placement. The device will be worn for seven consecutive days without being removed (unless electrodes require replacing) and data will be analysed to determine the duration of sedentary behaviour (<1.5 METS), light intensity (1.5–2.9 METS), and moderately intense activity (≥3 METS) [88] in bouts of at least 1 minute and at least 10 minutes. Participants will be blinded to the device’s ability to record activity data.

### Subjective assessment of physical activity behaviour

Participants will complete the Baecke physical activity questionnaire as a measure of habitual physical activity over the previous 12 months [89]. Separate work, sport and leisure index scores will be calculated, plus a total score.

### Seven-day exercise recall

Participants will be interviewed during consultations prior to the commencement of the physical tests. The participant will be guided through a recall of the previous 7 days for engagement in exercise bouts of at least 10 minutes in duration. Intensity will be reported by the participant through description of effort, breathing or the use of the Borg scale for ratings of perceived exertion [90]. The weekly time spent exercising at moderate to vigorous intensity for bouts of at least 10 minutes will be used for analysis. This lower limit was set due to a lack of health benefits achieved at lower duration and intensities [91].

### Blood sampling and analysis

#### Fasting blood collection and analysis

Venous blood samples will be collected prior to the oral glucose tolerance test (OGTT) by a trained phlebotomist. Forty millilitres of blood will be extracted into vacutainer tubes, and two tubes will be sent to a commercial pathology laboratory for analysis of fasting glucose, insulin, blood lipids, haemoglobin A1c, and C-reactive protein. The remaining blood will be centrifuged and stored in a freezer at −80 °C. Markers of systemic inflammation and bone metabolism will be analysed as described previously [92, 93]. Each participant is advised to maintain their normal diet and exercise patterns for the 3 days prior to the testing. After a 12- hour overnight fast, participants will attend the Baker IDI Heart and Diabetes Institute. Baker IDI Heart and Diabetes Institute research colleagues will perform muscle sympathetic nerve activity recording, endothelial function assessment and the OGTT. An intravenous catheter will be placed in an antecubital vein for blood sampling. Volunteers will ingest 75 g of glucose over 2 minutes (Glucotol™, Orion Laboratories, Welshport, WA, Australia). Blood samples will be drawn at −15, 0, 30, 60, 90 and 120 minutes for determination of plasma glucose and insulin concentrations. The remaining blood will be centrifuged and plasma will be stored in a freezer at −80 °C at the Baker IDI Heart and Diabetes Institute. Markers of bone metabolism and systemic inflammation will be analysed as described previously [92] from the stored samples.

### Muscle sympathetic nerve activity (MSNA) recording

Recordings of multi-unit post-ganglionic MSNA will be made while the subject rests in a comfortable supine position. A tungsten microelectrode (FHC, Bowdoinham, ME, USA) will be inserted directly into the right peroneal nerve at the fibular head by an experienced investigator (EL). During MSNA recording, blood pressure (BP) will measured continuously using the Finometer system (Finapress Medical System BV, Amsterdam, The Netherlands) and heart rate (HR) will be extracted from a three-lead ECG. All of these parameters will be digitised with a sampling frequency of 1000 Hz (PowerLab recording system, model ML 785/8SP, ADInstruments, Bella Vista, NSW, Australia). Resting measurements will be recorded over a 15-minute period and averaged. Sympathetic bursts will be counted manually and expressed as burst frequency (bursts/minute) and burst incidence (bursts/100 heart beats).

### Twenty-four-hour ambulatory blood pressure monitoring

Participants will be fitted with ambulatory blood pressure monitoring equipment (Oscillometric monitor, model number 90207, SpaceLabs Medical Inc., WA, USA) before leaving the Baker IDI Heart and Diabetes Institute on the first day at each assessment timepoint. The device will be worn around the waist with a belt while the cuff will be fitted to the upper left arm to measure brachial blood pressure every 15 minutes between 6 am and 10 pm, and every 30 minutes between 10 pm and 6 am. Participants will be advised to record their activity and any symptoms at each reading. The monitor is removed after 24–26 hours of continuous wear and then returned via a self-addressed express post satchel or in person to the Baker IDI Heart and Diabetes Institute. Blood pressure values will be averaged over the total recording time for a 24-hour average, and separately across waking and sleeping hours [94]. Resting blood pressure will be also recorded in the clinic after sitting quietly for 5 minutes. Blood pressure will be taken by an experienced researcher using a standard aneroid sphygmomanometer, listening for the first and last Korotkoff sounds through the brachial artery with a stethoscope.

### Spontaneous cardiac baroreflex function

Baroreflex sensitivity will be assessed using the sequence method from analysis of the continuous (beat-to-beat) blood pressure and heart rate fluctuations. The procedure identifies the ‘spontaneous’ sequences of three or more consecutive beats in which systolic blood pressure progressively rise (by at least 1 mmHg) and cardiac interval lengthens, or systolic blood pressure progressively falls (by at least 1 mmHg) and cardiac interval progressively shortens, with a lag of one beat. For each sequence, the linear correlation coefficient between cardiac interval and systolic blood pressure will be computed and the sequence validated when *r* >0.80. The slope between cardiac interval and systolic blood pressure will be calculated for each validated sequence and an average slope will be calculated for each recording. These measures will be extend by including assessment of baroreceptor modulation of MSNA (or vasomotor tone) by relating each spontaneous sympathetic burst to the diastolic blood pressure and cardiac interval of the heart beat during which the burst is generated [95].

### Endothelial function

The Endo-PAT 2000 will be used to non-invasively assess endothelial function and arterial stiffness. The device captures a beat-to-beat plethysmographic recording of the finger arterial pulse wave amplitude with pneumatic probes, detecting the peripheral arterial tone. Peripheral arterial tonometry is assessed in response to reactive hyperemia by placing the finger probe on the index finger in each hand (occluded study hand, and non-occluded, control hand). Measurements will be obtained for 5 minutes at baseline followed by 5 minutes of occlusion of one arm, with cuff inflated on the upper arm to supra-systolic pressure and then released to induce reactive (flow-mediated) hyperaemia, measured for 5–10 minutes. The magnitude of flow-mediated hyperaemia is calculated as the ratio between post- obstructive and baseline pulse wave amplitude, corrected to systemic changes measure in the contralateral, non-obstructed arm. Arterial stiffness is assessed by automatic calculating the Augmentation Index from the pulse arterial tonometry waveform.

### Quality of life questionnaires

The Medical Outcomes Trust (MOT) SF-36 (short form 36) health-related quality of life questionnaire contains 36 questions regarding the multi-dimensions of health. Question categories include: limitations of activities, general health, physical health problems, emotional health problems, social activities, pain, and energy and emotions [96, 97]. The questionnaires will be printed and provided to participants to complete at each timepoint.

### Multi-dimensional Body-Self Relations Questionnaire

The Multi-dimensional Body-Self Relations Questionnaire is a validated questionnaire [98] and the Appearance Orientation (AO) and Appearance Evaluation (AE) subscales have previously been used in this patient population [99]. AO quantifies the importance placed on appearance and grooming, and is scored on five-point responses (definitely disagree to definitely agree) to 12 questions. The AE score is comprised of seven items (again with five-point responses) and describes the participant’s perception of their own attractiveness.

The Work Productivity and Activity Impairment Questionnaire (WPAI) is a short self-report instrument that requires the participant to recall their work practices over the past 7 days. It has been used previously to assess work productivity loss and the degree of impairment to activity due to obesity and obesity-related co-morbidities [100].

### Epworth Sleepiness Scale

The Epworth Sleepiness Scale assesses daytime sleepiness during various situations on a four- point scale from 0 (would never doze) to 3 (high chance of dozing) [101].

### Musculoskeletal Pain Questionnaire

Participants will complete an 11-point (0–10) Likert visual analogue scale (VAS) to report pain in nine regions around the body as described elsewhere [102].

### Self-efficacy questionnaires

The Exercise Self-efficacy Scale (ESE) measures perceived competence to overcome barriers to completing exercise. The five items on the scale require the participant to rate the statement from 1 (not at all confident) through to 7 (very confident). The second self-efficacy scale, the Weight Efficacy Lifestyle Scale (WEL), measures self-efficacy for appropriate eating. It has five subscales (negative emotions, availability, social pressure, physical discomfort, positive activities) with four items each. Each item presents a statement that is then ranked on a 10-point scale, 0 (not confident) to 9 (very confident) [103, 104].

### Beck Depression Inventory-II

The Beck Depression Inventory-II (BDI) [105] is a revision of the original 21-item questionnaire with scores ranging from 0 to 3 on a 4-point Likert-type scale for each question (responses ranging from ‘not at all’ to an extreme form of each symptom) used to assess severity of depressive symptoms over the past 2 weeks including the current day, in populations aged 13 years and over. This tool is not used for diagnostic purposes, rather it is used to identify and score the severity of symptoms of depression.

### Spielberger State and Trait Anxiety Inventory

The Spielberger State and Trait Anxiety Inventory (STAI) is used to measure the presence and severity of current symptoms of anxiety along with general tendency to be anxious via self-report [106]. This tool consists of two questionnaires of 20 items each, the first questionnaire is used to measure state anxiety (feelings of anxiety at the time of administration) while the second, measures trait anxiety (general feelings of anxiety) [107]. Each questionnaire uses a four-option response scale from ‘not at all’ to ‘very much so’ (scored from 1 to 4) in the state anxiety questionnaire and ‘almost never’ to ‘almost always’ (scored from 1 to 4) for trait anxiety [106]. Scores range from 20 to 80 for each questionnaire with higher scores indicate greater anxiety. A cut-point of 39 to 40 being set for clinically significant symptoms of state anxiety [108, 109].

### Statistical analysis

All variables will be plotted and visually inspected for skewness and kurtosis. Formal analysis of normality will be confirmed with the Kolmogorov-Smirnov test of normality (SPSS statistics for windows, version 21.0: IBM Corp., Armonk, NY, USA) as it is appropriate when used with small- to medium-sized samples [110]. The independent *t* test for continuous variables will be used to examine the groups (ER versus EXER) characteristics at baseline using Genstat statistical software (for Windows 16th edition. VSN International, Hemel Hempstead, UK). Genstat will be used to produce a histogram of residuals, fitted-value and normal plot of residuals and assessed for normality, independence, equal variance and linearity for each dependent variable. If the data is shown to not be normally distributed or displays heteroscedasticity the data will be log transformed to restore normality [111]. Factorial repeated measures analysis of variance (ANOVA) (mixed between-within subjects or split-plot) with intention-to-treat protocol for all variables to assess the interaction effect (group × time) will be performed. Missing values for variables will be replaced using an expectation maximisation algorithm with a multiple imputations method [112] using Genstat statistical software. Analysis for the least significant difference of the means will be performed for both time and group with a *p* value set at 0.05.

## Discussion

Previous research demonstrates various health benefits of including exercise training as part of a healthy lifestyle at all BMI ranges. Although clinical practice guidelines recommend exercise training as part of the first-line treatment for overweight and obesity, there are few studies that demonstrate the effectiveness of exercise in class II and class III obesity. The study aims to determine whether the addition of exercise training to a VLED provides more favourable improvements in body composition, physical function, quality of life, and markers of cardiometabolic risk for women with clinically severe obesity, compared to VLED alone. The results of this study should allow for a greater understanding of the benefits and limitations of exercise training for high risk class II and class III obese women during periods of energy-restricted weight loss. It is expected that this research will guide clinical practice in the primary care setting, so that clinicians can provide more informed evidence-based practice for the management of clinically severe obesity.

## Trial status

The EMPOWER trial is active with participant recruitment and intervention delivery currently ongoing.

## Abbreviations

ACSM: American College of Sports Medicine
AE: Appearance Evaluation
AO: Appearance Orientation
BMI: body mass index
DXA: dual energy X-ray absorptiometry
ECG: electrocardiography
ER: energy restriction group
EXER: exercise training plus energy restriction group
ESE: Exercise Self-efficacy Scale
HRR: heart rate reserve
MOT: Medical Outcomes Trust
MSNA: muscle sympathetic nerve activity
OGTT: oral glucose tolerance test
RM: repetition maximum
RPE: rating of perceived exertion
STS: Sit to Stand
VAS: visual analogue scale
VLED: very low energy diet
VO_2_: volume of oxygen
VCO_2_: volume of carbon dioxide
WEL: Weight Efficacy Lifestyle Scale
WPAI: Work Productivity and Activity Impairment
